# Effects of Telephone Follow-Up Intervention on %Body Fat, Inflammatory Cytokines, and Oxidative Stress in Obese Hispanic Children

**DOI:** 10.3390/ijerph16162854

**Published:** 2019-08-09

**Authors:** Hyun-Seung Rhyu, Kyung-Shin Park

**Affiliations:** 1 Major of Sports Coaching, Department of Physical Education, Jungwon University, Chungbuck 28024, Korea; 2 College of Nursing and Health Sciences, Texas A&M International University, Laredo, TX 78041, USA

**Keywords:** obesity, Hispanic children, summer camp, follow-up intervention, telephone intervention, inflammatory cytokines, oxidative stress

## Abstract

This study investigated whether 10 month telephone follow-up intervention effectively stabilizes reductions in %body fat, and markers of inflammation and oxidative stress obtained from summer camp in obese Hispanic children. Fifty-six obese children (19 SUTI: summer camp and 10 months of follow-up telephone intervention, 18 SU: summer camp intervention only, and 19 CON: no intervention) completed this study. Anthropometric data and blood samples were obtained before (PRE), after 8 weeks of summer camp, and a 10month follow-up telephone intervention to measure markers of inflammation and oxidative stress. Eight weeks of summer camp significantly reduced %body fat, and levels of tumor necrosis factor-alpha, C-reactive protein and 8-hydroxydeoxyguanosine. It also elevated levels of adiponectin and total antioxidant status in SUTI and SU (*p* < 0.05). However, results of the 10month follow-up measurement were reverted back to PRE in SU, whereas the results for SUTI remained different to PRE (*p* < 0.05). Results confirm that levels of inflammation and oxidative stress are correlated to changes in %body fat, indicating that fat loss is effective in preventing and managing obesity-associated disorders. It is suggested that a telephone intervention is an effective follow-up tool for stabilizing reductions in %body fat as well as levels of inflammation and oxidative stress that were obtained from an intensive summer camp program in obese Hispanic children.

## 1. Introduction

Obesity rates have increased dramatically in school-aged populations [1,2,3,4]. The poor health outcomes associated with obesity usually develop in the later stages of life; however, pathological processes begin in childhood [2,5]. The trends for obesity have shown a high prevalence in Hispanic children in the United States. Consequently, the alarmingly high incidence rate of obesity places this population at high risk for developing future health problems such as type 2 diabetes mellitus, dyslipidemia, atherosclerosis, and hypertension [3,4,6].

Chronic low-grade inflammation and oxidative stress are recognized as common causes of obesity-related disorders and characterized by increased pro-inflammatory cytokines [3,7] and oxidative stress [8,9,10,11]. It was reported that levels of pro-inflammatory cytokines and oxidative stress are higher in obese children, while adiponectin, an anti-inflammatory cytokine, is inversely associated with fat mass [2,9,11,12,13]. Several studies found that lifestyle intervention through dietary control and physical activities reduced markers of inflammation and oxidative stress in obese children along with fat loss [1,3,7,8,14,15,16,17,18], indicating that fat loss is effective in preventing and managing obesity-associated disorders.

Many studies have reported short-term success in the treatment of obesity; however, long-term success is not often recorded because subjects of short-term weight loss interventions have regained weight back after the termination of interventions [19]. Previous studies have employed the use of telephone intervention during follow-up after primary obesity intervention to prevent weight regain, but effects of the telephone follow-up intervention are still inconclusive [20,21,22]. Furthermore, none of these studies measured inflammatory cytokines and oxidative stress associated with obesity-related disorders.

From the previous findings, we presumed that subjects who completed an 8 week summer weight loss camp (summer camp) would show notable reductions in %body fat, and markers of inflammation and oxidative stress. Furthermore, we also hypothesized that a 10 month telephone follow-up intervention would effectively retain these reductions from the summer camp. Therefore, one of purposes of this study was to investigate changes in %body fat, and levels of inflammatory cytokines and oxidative stress after an 8 week summer camp and the primary purpose was to evaluate the effectiveness of a 10 month follow-up telephone intervention on maintaining improvements from an intensive summer camp in obese Hispanic children.

## 2. Materials and Methods

### 2.1. Subjects

A total of 65 subjects aged 10–14 years old (31 boys and 34 girls), from the Hispanic community, were recruited through local newspaper advertisements. Participants were considered eligible if their body mass index (BMI) was over the 85th percentile as determined by the CDC BMI percentile calculator for child and teen [23]. Eligible subjects for each gender were randomized into one of three groups in the order of registration. Compensation was paid to the subjects in control group instead of providing interventions written in the consent form. Number of subjects for each group was (1) control (CON, n = 21), (2) 8 week summer camp with 10 month telephone follow-up intervention (SUTI, n = 22), and (3) 8 week summer camp only (SU, n = 22). A total of 9 subjects were excluded due to missing more than 5 days of camp or and missing at least one data collection day. Therefore, a total of 56 subjects (19 CON, 19 SUTI, and 18 SU) completed this study. The experimental protocol was approved by the Institutional Review Board (TAMIU IRB 2011-04-05). Informed consent was obtained from all individual participants and their parents included in this study.

### 2.2. Summer Camp Intervention

The summer camp program was held five days a week between 13:00–17:00 for 8 consecutive weeks during summer break. Subjects participated in four 50-min training sessions every day: aerobic exercise, muscular strength and endurance, Zumba dance with stretching, and fun activities (such as tag games, modified ball games, dodge ball, basketball, etc.). A certified dietitian provided weekly diet plans to SUTI and SU during this 8 week period. The calorie intake for each subject was calculated using the equation for obese boys and girls aged 3–18 years [24].

#### 2.3. 10 Month Telephone Intervention 

After the 8 weeks of summer camp, a 10 month telephone follow-up intervention was only provided to subjects and their parents of the SUTI group. A pedometer (Omron, PA, USA) was provided to each subject in SUTI to calculate weekly steps during the 10 month telephone follow-up intervention. Those assigned to SUTI were encouraged through weekly phone calls to reach a goal of 10,000 steps/day, but it was not required. Parents reported weekly steps recorded in the pedometer and communicated with the investigators regarding any concerns in subjects’ diet and level of physical activity. No weekly diet plans were provided during this follow-up intervention.

### 2.4. Measurements

Subjects were required to visit the laboratory prior to attending the summer camp (PRE), immediately after camp (POST), and 10 months (1YEAR) after the end of camp. During each visit to the laboratory, the subjects had their physical characteristics measured as well as 5 mL of blood drawn for blood chemical analyses.

Subjects’ anthropometric measurements were obtained twice by a trained technician, with measurements of height and weight being calculated to the nearest 0.1 cm and 0.1 kg, respectively. During height and weight measurements, subjects wore indoor clothes and no shoes.

Percentage of body fat was determined using bioelectrical impedance analysis (BIA: Quantum IV, RJL systems, MI, USA). Subjects were required to have at least 10 h fasting and void their bladder before the BIA test to minimize measurement errors. During BIA measurement, subjects were asked to lie supine, with arms 30° from the body and legs not touching. Electrodes were placed on the dorsal surface of right hand and foot following the manufacturer’s guidelines. Resistance and reactance were measured to determine %body fa using the company provided software.

Five mL of venous blood was drawn from an antecubital vein right after %body fat measurement. Collected blood sample was centrifuged in serum separating vacutainer tubes at 1000× *g* for 15 min (Allegra X-15R Refrigerated Centrifuge, Beckman Coulter, Irving, TX, USA). The serum was then stored at −80 °C until blood chemical analyses were conducted.

Tumor necrosis factor alpha (TNF-α), C-reactive protein (CRP), adiponectin, total antioxidant status (TAS), and 8-hydroxydeoxyguanosine (8-OHdG) in blood were measured using the enzyme-linked immunosorbent assay. All these variables were determined with commercial kits (Cayman Chemical Co., Ann Arbor, MI, USA) using a microplate reader (EL 808, BioTek Co, Winooski, VT, USA). The mean intra-assay CVs were 6.9%, 5.3%, 7.6%, 5.1%, and 6.3% and Inter-assay CVs were 7.3%, 8.2%, 8.7%, 7.8%, and 7.5% for TNF-α, CRP, adiponectin, total antioxidant status, and 8-OhdG, respectively.

### 2.5. Statistical Analysis

Sample size was estimated using the operating characteristic curve [25]. Power calculations were conducted to assess the overall changes in %body fat within the group using results from Kelishadi et al. [6] which indicated that 15 subjects in each group were needed to detect notable changes in %body fat through an intervention program with 80% power at α = 0.05. All statistical analyses were conducted using Sigmaplot 13 (Systat Software, inc., San Jose, CA, USA). Since all variables in this study passed the Shapiro-Wilk normality test and Brown-Forsythe equal variance test, two-way analysis of variance (ANOVA) with repeated measures were used to analyze changes in %body fat, TNF-α, CRP, adiponectin, total antioxidant status, and 8-OhdG during the summer camp and also 10 month telephone follow up intervention. Tukey post hoc test was performed when appropriate. Statistical significance was accepted for all tests at *p* < 0.05.

## 3. Results

Changes in physical characteristics at PRE, POST, and 1YEAR are shown up in Table 1. There were no differences found among three groups at PRE. Changes in %body fat showed different pattern in all three groups. When compared to PRE a decrease in %body fat was observed at POST in both treatment groups (SUTI: *p* = 0.003, SU: *p* = 0.008), while %body fat in CON was increased at POST (*p* = 0.028). From POST to 1YEAR, the SU saw an increase in %body fat (*p* = 0.017), but SUTI did not show any change in %body fat, indicating SUTI successfully maintained the reduction in %body fat from 8 weeks of summer camp for another 10 months (PRE vs. 1YEAR: *p* = 0.016). Changes in BMI was correlated to those in %body fat in this study.

**Figure 1:**
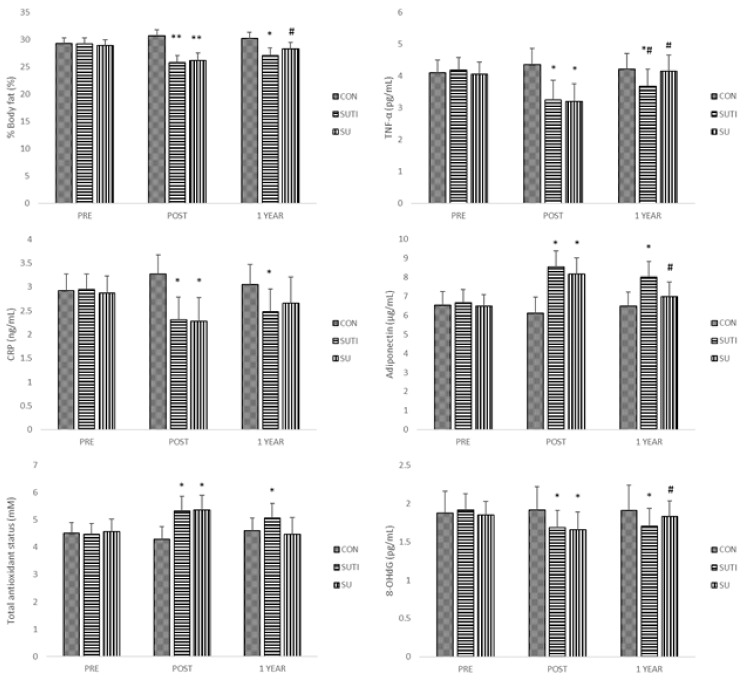
Changes in %body fat, markers of inflammation and oxidative stress at baseline (PRE), after 8 week summer camp (POST), and after 10 month follow-up (1YEAR). Values are means ± *SEM*. CON, control; SUTI, summer camp + 10 month telephone intervention; SU, summer camp only; TNF-α, tumor necrosis factor-alpha; CRP, C-reactive protein; *, different from PRE (*p* < 0.05); **, different from PRE (*p* < 0.01); #, different from POST (*p* < 0.05).

Changes in blood chemicals are displayed in Table 2 and Figure 1. Changes in levels of inflammatory cytokines were observed in SUTI and SU only. TNF-α decreased at POST as compared to PRE (SUTI: *p* = 0.02, SU: *p* = 0.016), which elevated in both treatment groups in 1YEAR (POST vs. 1YEAR, SUTI: *p* = 0.041, SU: *p* = 0.011); however, 1YEAR value in SUTI was still lower than PRE (*p* = 0.034). CRP at POST was lower than PRE in SUTI (*p* = 0.035) and SU (*p* = 0.031). At 1YEAR, only SUTI observed CRP lower as compared to PRE (PRE vs. 1YEAR: *p* = 0.043). Level of adiponectin was increased at POST in SUTI (*p* = 0.019) and SU (*p* = 0.024). This POST value was decreased by 1YEAR in SU (POST vs. 1YEAR: *p* = 0.047), while it was still greater than PRE in SUTI (PRE vs. 1YEAR: *p* = 0.038). Taken together, decreases in levels of TNF-α and CRP and an increase in adiponectin following an 8 week summer camp were observed in SUTI and SU. After the 10 month telephone follow-up intervention, SUTI maintained improvements in inflammatory cytokines from summer camp, while SU saw a reversion in all these variables almost back to PRE value. CON showed no change in any inflammatory cytokines.

Similarly to the changes in inflammatory cytokines, both SUTI and SU groups showed a reduction in oxidative stress after an 8 week summer camp. Level of total antioxidant status was increased at POST (SUTI: *p* = 0.024, SU: *p* = 0.027), while 8-OHdG was reduced at POST (SUTI: *p* = 0.017, SU: *p* = 0.021). SUTI maintained this improvement from summer camp over 10 months (PRE vs. 1YEAR, total antioxidant status: *p* = 0.039, 8-OHdG: *p* = 0.045); however, this improvement was reversed back to PRE in SU at 1YEAR (POST vs. 1YEAR, total antioxidant status: *p* = 0.011, 8-OHdG: *p* = 0.036).

## 4. Discussion

Previous studies noted an accelerated weight gain in children over the summer months compared to the school year [26,27,28], notably in Hispanic and African American children [29]. These results indicate that the prevention of weight gain over summer may have long-term health implications in children. Our findings correspond to the aforementioned studies as the control group in the present study observed an increase in %body fat over the summer break, while subjects who completed an 8 week summer camp found %body fat reduced. These results correlate with previous studies that have implemented the use of lifestyle intervention programs and found a reduction in %body fat [30,31].

Several studies reported decreases in markers of inflammation and oxidative stress following lifestyle interventions through dietary control and physical exercises [1,3,7,13,15]. A long-term intervention study for obese children observed a decrease in fat mass accompanied by reductions in pro-inflammatory mediators such as TNF-α and CRP and an increase of adiponectin [1]. Kelishadi et al. reported that %body fat, pro-inflammatory and oxidative states were reduced following 6 weeks of short-term lifestyle modification which included dietary advice and aerobic exercises [7]. Other studies also reported that weight reduction results in reductions of pro-inflammatory cytokines [3,14] and markers of oxidative stress [3,13] in obese children. Our results correspond with previous findings. The present study shows that subjects who completed an 8 week summer camp observed reductions in %body fat and levels of TNF-α CRP and 8-OHdG, as well as increases in adiponectin and total antioxidant status, while CON observed a slight increase in %body fat over the summer break with no changes in markers of inflammation and oxidative stress.

Although summer camps and other short-term obesity interventions have produced positive results, a study which conducted a follow-up measurement without secondary intervention reported that summer campers regained body mass and BMI in 10 months following the summer camp [19]. In the present study, results of %body fat and BMI in SU confirmed this finding. Telephone follow-up intervention was not provided to SU after their completion of the 8 weeks of summer camp. As the results, their weight loss through the summer camp was reverted back to pre-summer camp value, which was accompanied by reversions in levels of inflammatory markers and oxidative stress.

When used as the primary method in promoting short-term positive changes in obesity, the telephone intervention has exhibited beneficial results such as weight loss and increased physical activity [32,33,34]. However, as the follow-up intervention, effects of telephone calls are still inconclusive. A study carried out by Hyman et al. implemented a six-month telephone intervention following one-month randomized control trial and concluded that the telephone intervention was beneficial in maintaining changes in cholesterol and body weight [22]. Schiel et al. also implemented a 12 months of telephone/email intervention following a hospitalized weight reduction program for obese children and reported that subjects observed a weight loss from the primary intervention and weight stabilization during the telephone follow-up intervention [35].

In contrast, two studies with a long-term telephone follow-up intervention observed no differences in body weight and level of physical activity between telephone intervention and control groups [20,21]. Wing et al. utilized a 12 month telephone follow-up intervention after a six-month obesity intervention and found body weight after telephone follow-up intervention was still lower as compared to PRE, but also found no difference between control and intervention groups in the pattern of weight regain [20]. Ströbl et al. who also implemented a telephone follow-up intervention to morbidly obese adults, reached a similar conclusion, in which body weight and level of physical activity were stabilized during the telephone intervention, but no difference was found between the telephone intervention and control groups [21]. It is suggested that the maintenance of body weight and level of physical activity over the telephone intervention in both intervention and control groups might be due to another factor such as women’s pursuit of thinness or greater self-motivation to lose weight due to morbid obesity [36].

To our knowledge, this is the first study to measure changes in inflammatory cytokines and oxidative stress after a telephone follow-up intervention preceded by an intensive summer weight loss camp. In the present study, those who received the 10 month telephone follow-up intervention (SUTI) exhibited maintenance in %body fat, levels of inflammatory cytokines, and markers of oxidative stress from POST. The non-telephone intervention group (SU) observed a notable reversion to pre-camp values, thus strengthening the suggestion that the use of telephone intervention is effective in preventing long-term regression after the primary intervention terminated. Since none of studies investigated changes in levels of inflammatory cytokines and oxidative stress in the follow-up measurement we could not compare our results directly to previous findings. However, indirect evidence suggests that it is plausible to predict changes in inflammatory cytokines and oxidative stress from %body fat and body weight [37,38]. These two studies reported that subjects with greater inflammation gained more weight in follow-up measurements ranging from 3 to 9 years, indicating levels of inflammation is correlated to weight gain. Studies with children also reported that levels of inflammatory markers and oxidative stress were greater in overweight and obese children [2,3,39] and these variables decreased with weight loss [1,8,14,15]. Thus, it is reasonable to speculate that levels of inflammatory markers and oxidative stress are associated with changes in fat mass. The present study confirmed this speculation by showing that levels of inflammation and oxidative stress changed along with changes in %body fat and BMI.

The roles of chronic inflammation and oxidative stress on obesity-related disorders has been well demonstrated in recent studies [3,7,8,10]. It is known that obese children tend to become obese adults [40] and the rates of morbidity and mortality are greater in adults who were obese children, regardless of their adult body weight [41,42,43]. Since obesity is associated with various risk factors such as levels of chronic inflammation and oxidative stress [2,3,39], a combination of a successful obesity treatment and a long-term preventive intervention in childhood may reduce incidence of obesity-related disorders such as cardiovascular diseases, dyslipidemia, and type 2 diabetes mellitus in adulthood.

## 5. Conclusions

Implementing a secondary intervention is important in continuing obesity-related improvements over a long period of time, as improvements following short-term interventions are frequently reversed when the intervention is over. Our findings suggest that the telephone follow-up intervention is beneficial for school-aged children, as its low cost and ability to reinforce intervention strategies allow subjects to maintain their dietary adherence and physical activity throughout the school year. Furthermore, because primary interventions are too intensive to conduct during the school year, implementing a telephone follow-up provides school-aged children with a non-exhaustive approach to maintaining positive health changes that are seen after primary weight loss intervention.

There are two potential limitations to this study. Firstly, because the follow-up intervention was done through telephone, parents and subjects were simply asked whether they kept up with the recommended diet and pedometer steps. Therefore, there was difficulty in strictly controlling diet and exercise as subjects’ responses regarding diet goals and pedometer readings might not have been relayed to us accurately and truthfully. Secondly, because our study was conducted in a predominantly Hispanic area, it cannot be fully concluded whether telephone intervention as a follow-up to a summer weight loss camp is effective in maintaining positive results in other demographics. As a result, future research that administers the use of a telephone follow-up intervention should do so with strict diet and exercise control to ensure the effectiveness of telephone follow-up intervention. Additionally, future telephone follow-up studies should be conducted in areas with diverse demographics to assess differences in results between varying groups of individuals.

## Figures and Tables

**Table 1:** Physical characteristics of subjects at baseline (PRE), after 8 week summer camp (POST), and after 10 month follow-up (1YEAR).

	CON (n = 19)			SUTI (n = 19)			SU (n = 18)		
	PRE	POST	1 YEAR	PRE	POST	1 YEAR	PRE	POST	1 YEAR
Age (year)	12.2 ± 0.34			12.0 ± 0.33			12.1 ± 0.31		
Height (cm)	151.5 ± 2.4	151.8 ± 2.5	** 155.1 ± 2.8	152.3 ± 2.4	152.5 ± 2.4	** 156.1 ± 2.6	151.5 ± 2.3	151.7 ± 2.4	** 155.5 ± 2.7
Weight (kg)	62.5 ± 2.2	* 64.7 ± 2.4	**^#^ 66.9 ± 2.9	63.0 ± 2.5	* 60.7 ± 2.5	# 64.4 ± 3.3	62.0 ± 2.5	* 60.2 ± 2.5	*^##^ 65.9 ± 3.3
BMI	27.2 ± 0.5	* 28.1 ± 0.6	27.8 ± 0.6	27.2 ± 0.5	** 26.1 ± 0.5	*^&^ 26.4 ± 0.5	27.0 ± 0.5	* 26.2 ± 0.5	27.2 ± 0.6
%Body fat	29.3 ± 1.0	* 30.7 ± 1.1	30.2 ± 1.1	29.2 ± 1.1	** 25.8 ± 1.3	*^&^ 27.1 ± 1.3	28.9 ± 1.0	** 26.1 ± 1.4	^#^ 28.3 ± 1.2

Note: Values are means ± *SEM*. CON, control; SUTI, summer camp + 10 month telephone intervention; SU, Summer camp only; *, different from PRE (*p* < 0.05); **, different from PRE (*p* < 0.01); ^#^, different from POST (*p* < 0.05); ^##^, significantly different from POST (*p* < 0.01); ^&^, different from SU at 1YEAR.

**Table 2:** Changes in blood chemicals at baseline (PRE), after 8 week summer camp (POST), and after 10 month follow-up (1YEAR).

	CON (n = 19)			SUTI (n = 19)			SU (n = 18)		
	PRE	POST	1 YEAR	PRE	POST	1 YEAR	PRE	POST	1 YEAR
TNF-α (pg/mL)	4.10 ± 0.39	4.36 ± 0.49	4.21 ± 0.49	4.18 ± 0.40	* 3.24 ± 0.62	*^#^ 3.68 ± 0.52	4.05 ± 0.38	* 3.19 ± 0.56	^#^ 4.14 ± 0.51
CRP (mg/L)	2.92 ± 0.35	3.27 ± 0.41	3.05 ± 0.43	2.95 ± 0.33	* 2.31 ± 0.47	* 2.48 ± 0.47	2.87 ± 0.36	* 2.28 ± 0.49	2.66 ± 0.55
Adiponectin (μg/mL)	6.54 ± 0.71	6.13 ± 0.84	6.49 ± 0.74	6.68 ± 0.69	* 8.55 ± 0.85	* 8.03 ± 0.80	6.48 ± 0.60	* 8.18 ± 0.85	^#^ 6.98 ± 0.79
TAS (mM)	4.51 ± 0.40	4.29 ± 0.47	4.6 ± 0.47	4.47 ± 0.40	* 5.33 ± 0.54	* 5.07 ± 0.54	4.57 ± 0.47	* 5.36 ± 0.54	4.48 ± 0.61
8-OHdG (ng/mL)	1.88 ± 0.28	1.92 ± 0.30	1.91 ± 0.33	1.92 ± 0.21	* 1.69 ± 0.22	* 1.71 ± 0.23	1.85 ± 0.18	* 1.66 ± 0.23	^#^ 1.83 ± 0.21

Notes: Values are means ± *SEM*. CON, control; SUTI, summer camp + 10 month telephone intervention; SU, Summer camp only; TNF-α, tumor necrosis factor-alpha; CRP, C-reactive protein; TAS, total antioxidant status; 8-OHdG, 8-hydroxydeoxyguanosine; *, different from PRE (*p* < 0.05); **, different from PRE (*p* < 0.01); ^#^, different from POST (*p* < 0.05).
